# Deguelin inhibits vasculogenic function of endothelial progenitor cells in tumor progression and metastasis via suppression of focal adhesion

**DOI:** 10.18632/oncotarget.3752

**Published:** 2015-04-27

**Authors:** Minh Phuong Nguyen, Dongjin Lee, Se-Hyung Lee, Hye-Eun Lee, Ho-Young Lee, You Mie Lee

**Affiliations:** ^1^ Research Institute of Pharmaceutical Sciences, College of Pharmacy, Kyungpook National University, Daegu 702–701, Republic of Korea; ^2^ College of Pharmacy, Seoul National University, Seoul, 151–742, Republic of Korea; ^3^ School of Life Sciences and Biotechnology, College of Natural Sciences, Kyungpook National University, Daegu 702–701, Republic of Korea

**Keywords:** deguelin, endothelial progenitor cells, tumor vasculogenesis, focal adhesion, actin remodeling

## Abstract

Deguelin is a nature-derived chemopreventive drug. Endothelial progenitor cells (EPCs) are bone-marrow (BM)-derived key components to induce new blood vessels in early tumorigenesis and metastasis. Here we determined whether deguelin inhibits EPC function *in vitro* and *in vivo* at doses not affecting cancer cell apoptosis. Deguelin significantly reduced the number of EPC colony forming units of BM-derived c-kit+/sca-1+ mononuclear cells (MNCs), proliferation, migration, and adhesion to endothelial cell monolayers, and suppressed incorporation of EPC into tube-like vessel networks when co-cultured with endothelial cells. Deguelin caused cell cycle arrest at G1 without induction of apoptosis in EPC. In a mouse tumor xenograft model, tumor growth, lung metastasis and tumor-induced circulating EPCs were supressed by deguelin treatment (2 mg/kg). In mice tranplanted with GFP-expressing BM-MNCs, deguelin reduced the co-localization of CD31 and GFP, suggesting suppression of BM-derived EPC incoporation into tumor vessels. Interestingly, focal adhesion kinase (FAK)-integrin-linked kinase (ILK) activation and actin polymerization were repressed by deguelin. Decreased number of focal adhesions and a depolarized morphology was found in deguelin-treated EPCs. Taken together, our results suggest that the deguelin inhibits tumorigenesis and metastasis via EPC suppression and that suppression of focal adhesion by FAK-integrin-ILK-dependent actin remodeling is a key underlying molecular mechanism.

## INTRODUCTION

It is now clear that tumor blood vessel formation is not due exclusively to sprouting from pre-existing vessels. The *de novo* formation of blood vessels, called vasculogenesis, used to be thought to take place during embryonic development only. However, in the postnatal stage, endothelial progenitor cells (EPCs), which are bone marrow (BM)-derived precursors expressing endothelial cell marker proteins, enter the circulation in response to angiogenic factors, such as, vascular endothelial growth factor (VEGF) and stromal cell-derived factor (SDF)-1, target sites of neovascularization, and differentiate into endothelial cells, and thus, contribute to vessel formation [1]. Evidence now indicates that the term EPCs, endothelial colony forming cells (ECFCs) and colony forming unit-EC (CFU-EC) are used depending on their current methods of identifying or quantifying the EC lineage potential, and has similar phenotypes or properties that contribute to postnatal vasculogenesis, particularly to the vasculogenesis associated with tumor progression [2, 3]. Therefore, it suggests that EPCs or ECFCs or CFU-ECs play an essential role in tumor development and metastasis [4, 5] and that these cells be viewed as a potential target in cancer. Furthermore, it is already refined EPC-colony-forming units (CFUs) for their heirachical relationship between primitive small-CFUs and definite large-CFUs *in vitro* [6] and we adpoted this method and consider these-CFUs as functional EPCs [2, 6, 7].

The plant-derived rotenoid, deguelin has been reported to be a strong cancer chemo-preventive agent that can suppress the growth of a number of cancers [8–11]. On the molecular level, deguelin inhibits phosphatidylinositol 3-kinase (PI3K)/Akt signaling pathways. In particular, it has been reported: i) to suppress IκBα kinase activation, and thus, to suppress NFκB-regulated gene expression, potentiate apoptosis, and inhibit cellular invasion [12], ii) to inhibit Akt-mammalian target of rapamycin (mTOR)-survivin mediated cell survival [13], and iii) to induce p53-dependent apoptosis [9]. Other molecular effects attributed to deguelin include; the inhibition of mitochondrial bioenergetics [14], the inhibition of cyclooxygenase-2 expression [9], the induction of cell cycle arrest and apoptosis via regulation of the phosphorylation of Rb [11], and the transcriptional regulation of ornithine decarboxylase [15, 16]. Recently, several studies have described the effectivenesses of natural agents in terms of tumor control via EPC function inhibition [17, 18].

Focal adhesion plays a critical role as centers that transduce signals by cell-matrix interactions and regulate biological processes including proliferation, migration, and differentiation [19]. Focal adhesion kinase (FAK) is a non-receptor tyrosine kinase that is upstream regulator of multiple signaling pathways involved in cell adhesion, motility, survival and cell cycle progression [20]. By transducing signals to Rho family of small GTPases, FAK controls the dynamics of actin filament-based structures lamellipodia, filopodia, stress fibers and focal adhesions, which are crucial for cell adhesion and movement [21–23]. Binding of SH2 domains of p85 subunit of PI3K to autophosphorylated FAK activates PI3K-Akt survival pathways. FAK protects cells from apoptosis by activating the NFκB and mitogen-activated protein kinase (MAPK) pathways [24] and causing the degradation of p53 [25]. Enhanced expression of cyclin D1 and repression of p21 by FAK promote cell cycle progression from G1 into S phase [20, 26]. Integrin-linked kinase (ILK) is important regulator of EPC function via upregulation of stem cell-derived factor (SDF)-1 and intercellular adhesion molecule (ICAM)-1 [27]. Interestingly, both FAK and ILK collaborates with integrins for their downstream signal transduction, such as migration and proliferation [20, 21].

In this study, we examined the microscopic and molecular effects of deguelin on EPC function in tumor vasculogenesis and metastasis, with an involvement of focal adhesion of EPCs via FAK-integrin-ILK activation.

## RESULTS

### Deguelin inhibited the proliferation and the colony forming ability of EPCs

**Figure 1:**
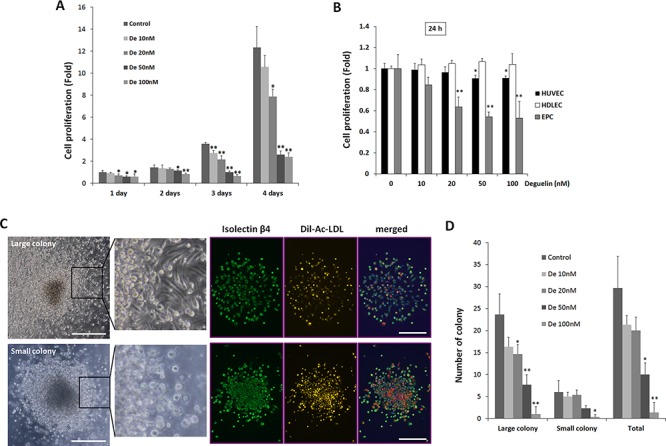
Dose-dependent suppression of EPC proliferation and colony formation by deguelin **A.** KSL cells (10.000 cells/well) were cultured in StemSpan media containing different concentrations of deguelin (*n* = 4/group). Relative cell numbers were measured by a CCK-8 assay at the indicated time points (*P < 0.05; **P < 0.001 *vs*. non-treated control). **B.** HUVECs, HDLECs were cultured in the EGM-2 media in different concentration of deguelin and relative cell numbers were measured by a CCK-8 assay at 24 h in compared to cultured EPCs as shown in A (*n* = 4). **C. D.** KSL cells were seeded in methylcellulose containing media containing deguelin at different concentrations (*n* = 4/group). Colony forming units were counted by blind investigators 12 days after seeding. Small- and large-CFUs were defined as focused clusters of rounded cells or as central core of round cells with elongated sprouting cells at their peripheries, respectively (left panel). Enlarged images of the boxed regions are shown in right. DiI-Ac-LDL was uptaken by these colonies and colonies were treated with FITC-conjugated anti-isolectin B4 antibody. **C.** Quantitative number of colonies were counted and graphed **D.** Three indepedent experiments were performed. *P < 0.05; **P < 0.01 *vs*. non-treated control. Scale bar = 500 μm.

The effect of deguelin on EPC proliferation was assessed by treating ‘cultured EPCs’ (similar to ECFC) from BM-derived c-Kit^+^/Sca-1^+^/lineage^−^ (KSL) cells with deguelin at different concentrations. Deguelin inhibited EPC proliferation in a dose-dependent manner (Figure 1A). Because deguelin exibits antiangiogenic activity in cancers [28], we tried to compare the effect of deguelin on EPCs with human umbellical vein endothelial cell (HUVEC) and human dermal lympatic endothelial cell (HDLEC). Deguelin inhibited prolfieration in HUVECs 10% at 50 and 100 nM, but not in HDLECs. However, prolfieration of EPCs was dramatically inhibited by deguelin 50–60% dose-dependently (Figure 1B). In semisolid media, EPCs formed two types of colonies, that is, small (primitive) or large (definitive) colonies, indicative of high proliferative activity and vasculogenic activity [6, 29]. In our colony forming assay (CFA), small- and large-CFUs were identified by their morphology and staining with Ac-LDL and isolectin β4 (Figure 1C). Furthermore, we characterized these CFUs for the expression of additional EC marker gene, such as VEGF receptor 2 (VEGFR2), CD31, endothelial nitrogen oxide synthase (eNOS), VE-cadherin, and vWF [7], and these-CFUs are deemed as functional EPCs, because these CFUs did not express myeloid cell markers, such as CD45 [6]. Fewer small and large colonies were formed in the presence of deguelin (Figure 1D), which suggested that deguelin attenuates both the self-renewal and differentiating abilities of EPCs.

### Deguelin induced cell cycle arrest at G1 phase and apoptosis in EPC

**Figure 2:**
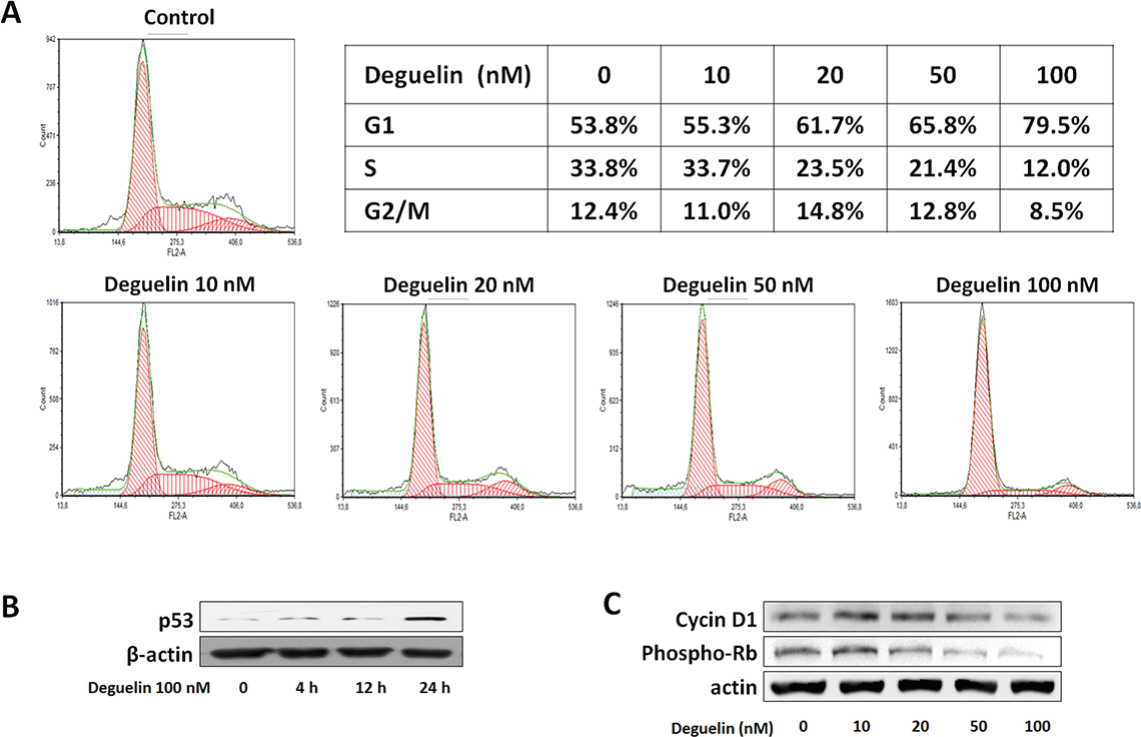
Regulation of cell cycle genes by deguelin in EPCs **A.** KSL cells were treated with different concentrations of deguelin for 2 days (*n* = 3). Cells were collected, fixed, and stained with propidium iodide. DNA contents were measured by flow cytometry and cell cycles were analyzed (*n* = 3). The effects of deguelin on the expression of p53. **B.** cyclin D1, and phosphorylated-Rb. **C.** β-Actin was used as the internal control (*n* = 3).

In a previous study, deguelin was found to cause apoptosis in several cancer cell lines, due to its inhibition of Akt survival pathways, but was not found to have apoptotic activity in endothelial cells [28]. In the present study, we examined the cytotoxic effect of deguelin in EPCs using an Annexin-V assay. Treatment with deguelin (100 nM for 24 h) did not significantly increase EPC apoptosis (Supplementary Figure 1), but cell cycle analysis showed that deguelin caused cell cycle arrest at G1. In fact, deguelin dose-dependently increased the number of cells in G1, decreased the number in the S phase, and had a minimal effect on the G2/M phases (Figure 2A). p53 mediates inhibition of G1 cyclin kinase and arrest cell cycle in G1 phase. Phosphorylation of pRb induces E2F-mediated gene transcription involved in cell cycle progression at the G1-S transition [30]. Thus we checked level of p53 and phosphorylated pRb protein. In these treated cells, the expression of p53 was increased (Figure 2B), and the expressions of cyclin D1 and of phosphorylated pRb were found to be suppressed (Figure 2C). These results indicate that deguelin inhibits EPC proliferation by regulating cyclin D1 and pRb-dependent cell cycle progression without any induction of apoptosis.

### Deguelin inhibited EPC function

**Figure 3:**
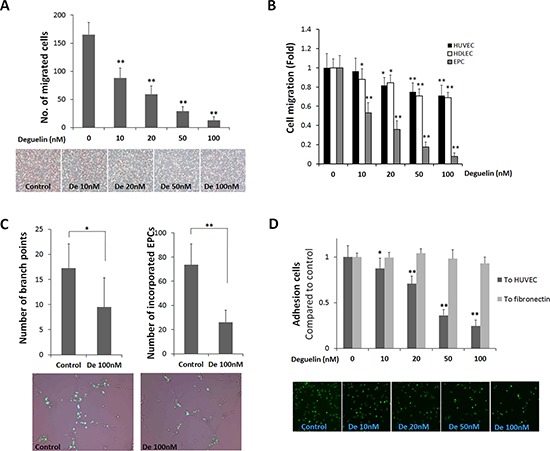
Effects of deguelin on EPC migration, adhesion, and tube formation **A.** A modified Boyden chamber assay was used with rhVEGF (20 ng/ml) as chemoattractant. EPC migration was identified as a red-purple color by staining with H&E after removing non-migrated cells (*n* = 4). **B.** The same migration assay experiment as in A using HUVEC, HDLEC, and cultured EPCs were performed in the presence or absence of deguelin (10–100 nM) in lower or upper chamber for 5 h (*n* = 3). **C.** GFP-expressing EPCs were co-cultured with HUVECs on Matrigel matrix for 8 h. Branching points and incorporated GFP-expressing EPCs were counted and graphed (*n* = 3). Representative pictures showing the incorporation of GFP-expressing EPCs into tube-like structures shown in below. **D.** EPCs were allowed to adhere to HUVEC monolayers for 3 h or to fibronectin for 30 min. The graph shows fold inductions of numbers of adherent cells versus non-treated control (*n* = 3). The lower panel shows representative pictures illustrating the adhesion of GFP-expressing EPCs to HUVEC monolayers. *P < 0.05; **P < 0.001 compared to control.

Next, we examined the effects of deguelin on the functional activities of EPCs, that is, migration, tube formation, and adhesion. In a modified Boyden chamber migration assay, deguelin significantly and dose-dependently reduced the number of migrating EPCs (Figure 3A). When compared with HUVECs and HDLECs, EPCs was the most susceptible to deguelin (Figure 3B), suggesting that EPCs can be vascular target cells for deguelin. Because the migration assay was performed in a short time (5 h), the observed effect of deguelin on EPC migration is substantially independent of its effect on cell proliferation.

The functional ability of EPCs to incorporate and differentiate into vascular tubes was examined on a Matrigel. EPCs from GFP transgenic mice were co-cultured with HUVECs on Matrigel matrix (100 nM). It was found that deguelin at 100 nM reduced the number of EPCs that were incorporated and the number that differentiated into tubular structures with HUVECs (Figure 3C), as determined by detecting GFP^+^ cells. However, using only HUVECs or HDLECs, deguelin had no effect on the tube formation (Supplementary Figure 2) as found in a previous study that deguelin at 100 nM does not alter the organization of HUVECs into capillary-like networks [28], and thus, the impaired tube formation observed was probably the result of inhibiting the ability of EPCs to incorporate and enhance tube-forming ability of endothelial cells.

Because the adhesion of EPCs to endothelial cells and the extracellular matrix is a crucial step for EPC docking and migration into a new site for EPC growth and to form new blood vessels [31], adhesion assay was performed. When EPCs from GFP-expressing mice were plated onto fibronectin-coated 96-well plates, numbers of adherent cells were unaffected by deguelin (Figure 3D). However, when EPCs were allowed to adhere to HUVEC confluent monolayers, deguelin reduced the number of adherent cells after 3 h (Figure 3D), which suggested that deguelin might inhibit EPC adhesiveness at sites of vasculogenesis.

### Deguelin inhibited tumor vascularization *in vivo*

**Figure 4:**
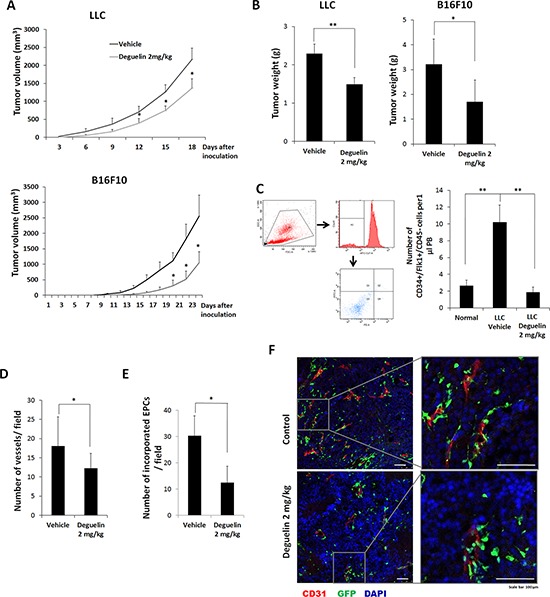
Effect of deguelin on tumor growth and vasculogenesis *in vivo* **A.** Mice were inoculated s.c. with LLCs or B16F10 cells and treated with deguelin (2 mg/kg body weight, *n* = 6) or vehicle (*n* = 6) every 2 days, and measured tumor volume. **B.** Tumor final weights of **A. C.** MNCs were isolated from peripheral blood by gradient centrifugation using Histopaque 1083 and stained with Alexa Fluor 647-conjugated anti-CD45, PE-conjugated anti-Flk-1 and FITC-conjugated anti-CD34 antibodies (*n* = 3). Cells were analyzed by flow cytometry. Debris and platelets were excluded by gating low FSC/SSC fractions. Circulating EPCs were defined as CD34^+^/Flk-1^+^/CD45^−^ cells. **D–F.** Mice transplanted with BM-MNCs from GFP-expressing mice were s.c. inoculated with B16F10 cells and treated with deguelin as above (*n* = 6). Tumors were fixed and subjected to IHC. Staining for CD31 revealed tumor micro-vessel densities. **D.** Among the tumor microvessels, GFP-expressing EPC-derived vessels were counted. **E.** CD31 (red) and the differentiation of GFP-expressing EPCs into endothelial cells (green) were shown. **F.** Enlarged images of the boxed regions are shown in right. Each experiment was performed twice independently. *P < 0.05; **P < 0.005; ***P < 0.001. Scale bar = 100 μm.

Mouse tumor cells, LLC and B16F10 xenograft models were used to examine the effect of deguelin on tumor vasculogenesis *in vivo* (tumor cells were injected s.c. into flanks). We chose 2 mg/kg of deguelin (i.p) which was 2~4-fold lower than those to inhibit cancer cell growth or metastasis [32, 33]. When animals were treated with 2 mg/kg of deguelin (i.p) every 2 days for 2 weeks, tumor growth was significantly reduced (Figure 4A) and final tumor weight was lighter than in vehicle control (50% DMSO in physical saline, i.p.) (Figure 4B). To determine the effect of deguelin on the number of circulating EPCs, in two weeks after tumor cell inoculation, peripheral blood was collected to assess circulating EPC numbers. Numbers of circulating EPCs (defined as peripheral blood MNCs positive for both CD34 and VEGFR2 but negative for CD45 [34]) were significantly elevated in tumor bearing mice, but not in deguelin-treated tumor bearing mice (Figure 4C), suggesting that low dose deguelin (2 mg/kg) inhibits tumor growth by suppressing vasculogenesis through inhibition of EPC mobilization from BM into circulating blood.

To explore this suggestion, after γ-ray treatment to deplete BM cells, mice were transplanted with BM-MNCs from GFP transgenic mice. These mice were then inoculated with B16F10 cells and treated with deguelin or vehicle. Two weeks later, tumor masses were collected and subjected to IHC. Staining for CD31 showed that tumor vessel densities were significantly reduced in deguelin-treated mice (Figure 4D and 4E), and that vessel sizes in treated mice were also smaller than control (Figure 4F). In particular, the co-expressions of CD31 and GFP (indicative of the differentiation of BM-derived EPCs into vascular endothelial cells) were reduced in the tumors of deguelin-treated mice (Figure 4E and 4F). These findings suggest that deguelin inhibits the mobilization of EPCs from BM into the peripheral blood circulation and their recruitment and incorporation into tumor neovasculature.

### Deguelin inhibited cancer cell metastasis through suppression of EPC function

**Figure 5:**
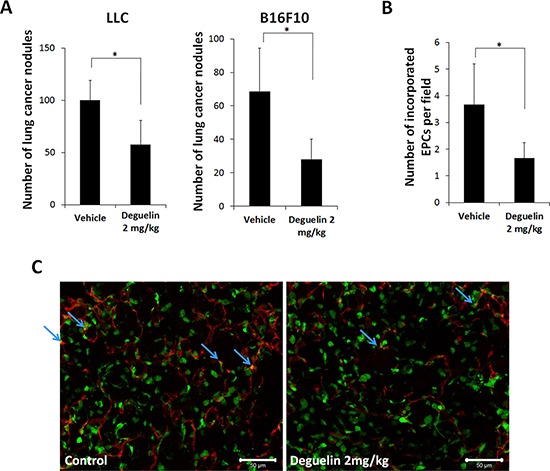
Deguelin inhibited metastasis Mice transplanted with BM-MNCs from GFP-expressing mice were inoculated i.v. with 4 × 10^4^ LLCs or 10^5^ B16F10 cells and treated with deguelin (2 mg/kg) or vehicle (*n* = 6/group) every 2 days). **A.** Numbers of lung metastases after 12 days (*P < 0.01). **B, C.** IHC staining of lung nodule sections for CD31 (red) and GFP (green) revealed vessel densities and the incorporation of EPCs (arrows). *P < 0.05. Scale bar = 100 μm.

As EPCs play an important role in metastasis progression [4] and deguelin inhibited EPC function from our results, we investigated whether deguelin inhibits metastasis and EPC contribution to metastasized tumor. BM transplanted mice with GFP-expressing MNCs were inoculated with LLC or B16F10 cells intravenously and treated with deguelin or vehicle every two days for 12 days. At 2 weeks post-injection, lung metastasis was inhibited (Figure 5A), lung nodule vascularization was also reduced analyzed by the CD31^+^/GFP^+^ cells. Therefore, the contribution of EPCs or vascular endothelial cells in metastasized lung nodules was inhibited in deguelin-treated mice (Figure 5B and 5C).

### Deguelin inhibited ILK-integrin αvβ3-FAK signaling axis and focal adhesion

**Figure 6:**
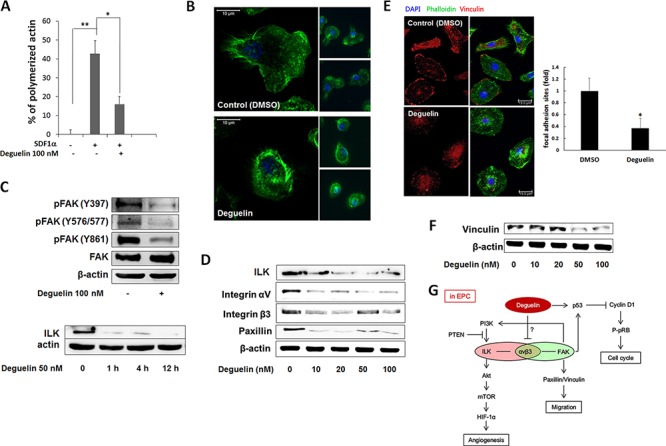
Deguelin suppressed focal adhesion via inhibition of FAK-integrin-ILK axis **A.** Freshly isolated BM-lin^−^-c-Kit+ cells were pre-incubated with 100 nM deguelin or vehicle for 1 h. Actin polymerization was induced by SDF-1α (200 ng/ml) for 20 sec. Cells were fixed and stained with phalloidin and c-Kit antibody, and analyzed by flow cytometry (*n* = 3). *P < 0.005; **P < 0.001. **B.** Cultured EPCs were treated with deguelin (100 nM) or vehicle (DMSO) for 1 h. Filamentous actin was visualized by immunofluorescent staining using Alexa Fluor 488-conjugated Phalloidin (*n* = 3). **C.** Cultured EPCs were treated with deguelin (100 nM) for 15 min. Total protein was extracted and western blotted for activated FAK (*n* = 3). **D.** Cultured EPCs were treated with deguelin (10–100 nM) for 15 min. Wetern blot analysis with total protein for ILK, integrin αV, β3, and paxillin was performed. β-actin was used for internal control. **E.** Cultured EPCs were treated with deguelin (100 nM) or vehicle (DMSO) for 1 h. Actin polymerization was visualized by immunofluorescent staining using Alexa Fluor 488-Phalloidin. Focal adhesion was immunostained with mouse anti-vinculin antibody and Alexa Fluor 488-phalloidin. Focal adhesion sites were counted and graphed. *P < 0.001 **F.** Cultured EPCs were treated with deguelin (10–100 nM) for 15 min. Wetern blot analysis for vinculin was performed. β-actin was used for internal control. **G.** A scheme for the molecular mechamism of deguelin against EPCs function in vasculogenesis.

Since actin polymerization in response to chemokines is a key event in cell cycle progression, cell migration, and tumor metastasis [35], we studied the effect of deguelin on the polymerization of actin within BM-derived lin^−^/c-Kit^+^ cells. Stimulation with SDF-1α induced actin polymerization within 20 sec, but pre-treatment with deguelin for 1 h strongly diminished SDF-1α-induced actin polymerization (Figure 6A). Furthermore, phalloidin immunofluorescence showed that treating cultured EPCs with deguelin disrupted cell polarity and focal adhesions. Cultured EPCs are polarized with broad lamellipodia extending in the direction of cell migration and a narrow uropod at the rear (Figure 6B, upper), which indicates migrating ability. However, this migratory morphology was disrupted after 1.5 h of treatment with deguelin when actin skeletons were stabilized with a dense meshwork of actin filaments around the cell periphery, which made cells contracted and depolarized (Figure 6B, lower). These findings suggest that the inhibitory effects of deguelin on EPC function are achieved via the suppression of actin polymerization and cytoskeletal remodeling. To investigate underlying molecular mechanism for this, molecules for cytoskeletal machinery were examined. The cytoskeletal network is under the control of the Rho family of small GTPases, which in turn, are regulated by FAK. The activation of FAK, a rapid event in response to growth factors or cell adhesion to ECM, is crucial for focal adhesion turnover and cell motility [22]. Western blot showed decreases in the phosphorylation of FAK at Tyr397, Tyr576/577, and Tyr861 in EPCs treated with deguelin (Figure 6C), which suggests that deguelin regulates migration and adhesion by actin remodelling through inhibiting FAK phosphorylation. ILK and FAK shares integrin αvβ3 for their signaling for actin remodeling, we checked ILK, integrin signaling and paxillin expression. Deguelin dose-dependently decreased ILK as well as integrin αvβ3 and paxillin (Figure 6D). Focal adehision number as determined by vincullin immunostaining in lamellipodia was significantly decreased by deguelin treatment (Figure 6E). And vinculin protein expression was decreased by deguelin (Figure 6F)

When we investigated whether Akt pathways are affected in EPCs, the VEGF-induced activations of Akt and mTOR were suppressed dose-dependently by deguelin in EPCs (Supplementary Figure 3A). In complete media, this pathway was inhibited at early time points (5 min-1 h) (Supplementary Figure 3B).

## DISCUSSION

The targeting of angiogenesis is a valuable strategy for the treatment of solid cancers, because it targets genetically stable cells rather than rapidly mutating and drug resistance acquiring cancer cells [36]. Because EPCs are required for tumor vascularization [37], the effectiveness of cancer therapy could be enhanced by targeting EPC growth and function. In addition, bone marrow-derived EPCs has been known as critical regulators of angiogenic switch in metastatic progression. EPCs homing to metastatic lesions induces tumor progression through direct luminal incorporation or secreting angiogenic cytokines by paracrine manner, and ultimately forms neovessels in metastatic tumors [4]. In particular, antitumor agents with EPC inhibitory activity can provide an additive treatment effect [38]. Deguelin is a potent anticancer agent, and inhibits angiogenesis by inhibiting endothelial cells [28]. In this study, we show that deguelin inhibits EPC function *in vitro*, and the contribution by EPCs to tumor growth and metastasis *in vivo*, through inhibiting actin remodeling and focal adhesion signaling.

To contribute to vascularization, EPCs must proliferate, mobilize from BM into the circulatory system, home to tumor sites, and differentiate into endothelial cells [39]. In particular, the proliferation, migration, adhesion, and differentiation of EPCs are required for tumor vascularization, growth, and metastasis [5]. Therefore, a combinatorial approach to the targeting these processes leads to a strongly inhibitory effect on EPC function during tumor progression.

The inhibition of cell growth by blocking Akt pathways is the main reported effect of deguelin [8, 28, 40], and in the present study, we observed this in EPCs (Supplementary Figure 3). However, whereas Akt suppression is implicated in the induction of apoptosis in almost all cancer cells examined, deguelin was not observed to have a cytotoxic effect on EPCs, despite its considerable inhibition of EPC proliferation. This finding is consistent with a lack of toxicity on non-cancer cells [28, 40]. Deguelin has even been reported to inhibit apoptosis significantly in endothelial cells [28]. Therefore, we can speculate that deguelin demonstrates its pharmacological effects depending on the different celluar context.

Cellular context-dependent effects of deguelin is also shown when it did not affect the proliferation of lymphatic endothelial cells, and just slightly reduced that of endothelial cells (Figure 1A). While strongly suppressing EPC functions, deguelin moderately inhibited migration of endothelial cells and lymphatic endothelial cells, and did not changes tube formation capacity of these cells (Figure 4B, 4D). Therefore, anti-angiogenic effect of deguelin *in vivo* might be primarily due to inhibition of EPC, rather than endothelial cell proliferation and functions.

There is still no uniform definition of EPCs. From our results, our small- or large-EPC-CFUs express Ac-LDL, isolectin B4 but did not express either CD14 or CD45, which represent similar phenotypes to ECFCs [3]. Role of EPCs in blood vessel formation have been demonstrated, as circulating EPCs can incorporate into sites of neovascularisation [41]. However, beside EPC identity, the extent of contribution of EPCs to tumor neovascularization remains controversial. While many investigations described great contribution of EPCs to tumor vessel formation [5] some others showed minimal [42] or even undetectable contribution [43]. These different reports can arise from differences in EPC markers and tumor models employed for analysis.

Given the rarity of EPCs in peripheral blood, vasculogenesis greatly depends on the abilities of EPCs to mobilize toward angiogenic factors and adhere to developing vasculature. In the present study, deguelin dramatically inhibited EPC migration and adhesion to HUVECs (Figure 3A, 3D). Furthermore, deguelin reduced tube formation in EPC-HUVEC co-culture assays (Figure 3C). EPCs have been reported to be incorporated into and to enhance capillary-like networks formed by endothelial cells [44]. In addition, in a previous report, deguelin at 100 nM did not alter HUVEC organization into capillary-like networks (Supplementary Figure 2) [28], the effect of deguelin on co-cultured tube formation demonstrates that deguelin suppresses the ability of EPCs to incorporate and stimulate endothelial cell tube formation, and suggests that it has an inhibitory effect on the functional differentiation of EPCs and thus EPCs a specific vascular target of deguelin.

Deguelin is an well-known Akt-inhibitor [40] and a HSP90 inhibitor in anticancer activity [32]. It can reduce stability of HSP90 sensitive client HIF-1α in cancer cells. As shown in cancer cells, inhibition of EPC function by deguelin may result in part from the suppression of Akt pathways (Supplementary Figure 3) and the hypoxia-induced expressions of VEGF and CXCR4 (Supplementary Figure 4). However, we found the *in vivo* suppressive effect on the tumor growth by deguelin was obtained at 2–4 fold lower dose than that of anticancer activity *in vivo* [32, 33]. At this concentration we cannot found the tumor cell necrosis or death (data not shown), suggesting that the inhibition of tumor cell growth is due to only suppressing EPC function.

In the present study, we found that deguelin caused cell depolarization and contraction. After treatment, cells lost their typical migratory morphology and exhibited dense actin filament meshes around their peripheries (Figure 6B). Furthermore, deguelin consistently inhibited the activation of FAK and SDF-1α-induced actin polymerization as well as expression of ILK, integrins and paxillin (Figure 6A, 6C, 6D). Activation of integrins by binding to specific extracellular matrices (ECM) activates FAK as well as ILK signalings. Signaling cascade of FAK activation is essential for cell migration [45], and regulates Rho family GTPases cdc42, Rac and Rho, which are responsible for the formation of filopodia, lamellipodia, and stress fibers, respectively [21, 46]. FAK also activates actin-dependent membrane ruffle and lamellipodia formation required for cell migration [23]. ILK interacts with paxillin and FAK and mediates cytoskeletal dynamics and cell migration [47]. From our findings that the inhibition of FAK and ILK by deguelin may results in decreased integrin signaling-mediated focal adhesion and migration as well as cell contraction. In addition, the effect of deguelin on activation of Akt and expression of p53 and cyclin D1, which are down-stream targets of FAK, suggests that the inhibition of FAK may also be responsible for the decrease of cell growth by deguelin. From our results, integrins αvβ3 were inhibited by deguelin (Figure 6), suggesting integrins αvβ3 are involved in FAK and ILK signalings in EPCs (Figure 6G). Interestingly, FAK is also a downstream target of several growth factor receptors [48] and acts at both upstream and downstream of Akt [49, 50]. Therefore, it is important to clarify if integrins αvβ3, Akt, FAK, or another upstream mediator is a direct target of deguelin (Figure 6G). With our data, deguelin suppresses migration, proliferation, angiogenesis activities in EPCs through FAK/integrin axis. Even we focused on deguelin's effect on FAK/ILK signaling pathway, VEGF or SDF-1α signaling pathway may be also invoved in the inhibition of vasculogenic function in EPCs by deguelin.

Summarizing the present study shows that deguelin, a potent anticancer drug, suppresses tumor growth and metastasis by inhibiting EPC function, and thus, inhibits tumor vasculogenesis. Furthermore, in the present study, it was found for the first time that deguelin inhibits actin filament remodeling.

## MATERIALS AND METHODS

### Mice

Animal experiments were performed using C57BL/6J mice (SLC, Japan), which were handled in strict compliance with the guidelines for care and use of laboratory animals issued by the institutional ethical animal care committee of Kyungpook National University (Daegu, Korea). Mice were maintained under specific pathogen-free conditions. Transgenic mice that universally expressed the green fluorescent protein (GFP) under the chick β-actin promoter were kindly provided by Dr. Goo-Taeg Oh in Ewha University.

### EPC isolation and culture

BM-derived mononuclear cells (MNCs) were isolated from 8-to-12-week old C57BL/6J mice by gradient centrifugation using Histopaque 1083 (Sigma Aldrich, St. Louis, MO). Remaining red blood cells were depleted with NH_4_Cl. BM-MNCs were cultured on 2% gelatin-coated dishes in EGM-2 media (Lonza, Basel, Switzerland) supplemented with 5% FBS and antibiotics. Non-adherent cells were removed and the medium was changed on day 4. Isolation and culture of c-Kit^+^/Sca-1^+^/lineage^−^ (KSL) cells from BM-MNCs were performed as described previously [51].

### EPC colony forming assay (CFA) and characterization

EPC colony formation was assessed by culturing 500 KSL cells in triplicate in methylcellulose-containing medium M3236 (Stem Cell Technologies) supplemented with; 100 ng/ml SCF (PeproTech), 50 ng/ml VEGF (PeproTech), 20 ng/ml interleukin-3 (R&D Systems), 50 ng/ml basic fibroblast growth factor (bFGF, R&D Systems), 50 ng/ml epidermal growth factor (EGF, PeproTech), 2 U/ml heparin (Sigma Aldrich), 30% FBS, and antibiotics. Small- and large-colonies (CFUs) after culture for 12 days were defined as focused clusters of rounded cells or as cell clusters with a central core of round cells and elongated sprouting cells at their peripheries [51]. Endothelial characteristics of the attached small- and large-CFUs were examined after uptake of DiI-conjugated Ac-LDL (DiI-Ac-LDL) (Biomedical Technologies Inc., MA) and binding with FITC-conjugated isolectin B4 (Sigma Chemical Co., WI), a standard marker of endothelial lineage cells. In our previous study, we defined these CFUs for the expression of additional marker genes such as CD31, Flk-1, eNOS [7], and in this study von Wilibrand factor (vWF), and VE-cadherin.

### Mice and bone marrow transplantation

C57BL/6J mice were housed under specific pathogen-free conditions in standard polycarbonate cages with stainless steel tops and micro-isolator lids with wood chip bedding. Ambient conditions were maintained at 21 ± 3°C and 30–60% RH under a 12/12 h light/dark cycle. Six-week old mice were exposed to a lethal dose (10 Gy) of ^60^Co radiation. Six hours later, mice were inoculated intravenously with 10^7^ BM-MNCs isolated from GFP transgenic mice. Four weeks after exposure, flow cytometry indicated that the frequency of GFP-expressing cells among the peripheral MNCs of bone marrow transplanted (BMT) mice was > 95%.

### Tumor growth, metastasis, and circulating EPC

At 4 weeks post-BM transplantation, mice were subcutaneously (s.c,) injected with 10^5^ Lewis lung carcinoma cells (LLCs) or B16F10 melanoma. Tumor volumes were measured every 2 days with a caliper. Three weeks after injection tumor masses were collected for immunohistochemistry (IHC). For the metastasis assay, BMT mice were inoculated intravenously (i.v.) with 4 × 10^4^ LLCs or 10^5^ B16F10. Twelve days later mice were sacrificed and lung nodules were counted.

### Actin polymerization assay

Freshly isolated BM-lin^−^ cells were incubated with deguelin or vehicle at 37ÐC for 1 h and stimulated with 100 ng/ml SDF-1α for 20 sec. Cells were then fixed with 4% paraformaldehyde (PFA), stained with Alexa Fluor 488-Phalloidin (Invitrogen), and PE-anti-cKit antibody (BioLegend), and analyzed with a FACSCalibur. C-Kit^+^ cells were gated to analyze filamentous actin contents.

### Proliferation assay

KSL cells (1 × 10^4^) were cultured in 96-well plates containing 100 μl StemSpan media supplemented with 100 ng/ml SCF (PeproTech), 50 ng/ml VEGF (PeproTech), 100 ng/ml IL6 (PeproTech), 100 ng/ml Flt3 (R&D Systems), 20 ng/ml TPO (PeproTech), and different concentrations of deguelin (10–100 nM) for 1–4 days. Cell Counting Kit-8 (Dojindo, Kumamoto, Japan) solution (10μl) was then added to each well and plates were incubated for a further 4 h and read for O.D. at 450 nm.

### EPC adhesion to matrix molecules

A 96-well plate was coated with 0.1 mg/mL fibronectin at 37ÐC for 3 h. Cultured EPCs were detached with 0.25% trypsin and resuspended in EGM2 media supplemented with 5% FBS. Cells (10^5^) were placed into each well and incubated for 30 min at 37ÐC. After removing non-adherent cells by washing three times, adhering cells were quantified using a 3-(4, 5-Dimethylthiazol-2-yl)-2, 5-diphenyltetrazolium bromide (MTT) assay.

### EPC adhesion to endothelial cell monolayers

Confluent monolayers of HUVECs were prepared on 4-well plates. EPCs expressing green fluorescence protein (GFP) were detached and 10^5^ cells were added to each well. After 3 h, non-adherent cells were removed by washing with PBS. Cells were then fixed with 4% paraformaldehyde, stained with DAPI, and counted in 5 random fields under a fluorescent microscope.

### Migration assay

EPC migration assays were performed in triplicate using a Transwell system (6.5-mm diameter, 5-μm pore size, Corning Costar, Cambride, MA). Six hundred microliters of EBM media with or without deguelin and 20 ng/ml rhVEGF were placed in the lower chambers. Cultured MNCs (in EGM2 containing 5% FBS for 7 days) were starved in FBS-free EBM media for 24 h, and seeded (5 × 10^4^ cells) into upper chambers in EBM media with or without deguelin. After incubation for 5 h, cells were fixed with methanol. Non-migrating cells on upper sides were scrapped off and the filter was stained with Hematoxylin and Eosin (H&E). Migrating cells were counted in five random high power fields (HPF) per membrane. Results are expressed as relative migrations (average number of migrated cells/HPF ± SD).

### Tube formation assay

Matrigel matrix (BD Biosciences) was thawed at 4°C overnight, and 70 μl was placed in a 96-well plate at 37°C for 30 min to solidify. GFP-expressing EPCs (1.5 × 10^3^ cells) and HUVECs (10^4^ cells) were co-plated in 100 μl EGM2 media and incubated at 37°C in 5% CO2 for 8 h. Wells were examined under a fluorescent microscope to assess capillary formation and GFP^+^ cell incorporation. Results are presented as the means ± SDs of numbers of branch points and numbers of EPCs incorporated into tube network in 5 HPFs.

### Apoptosis assay

KSL cells expanded for 7 days were subcultured for 24 h with deguelin (10–100 nM) or DMSO as a vehicle control in triplicate. Cells were then collected, washed, and double stained with FITC-conjugated Annexin V (Invitrogen, Carlsbad, CA) and propidium iodide (PI, Sigma Aldrich). Labeled cells (10^4^ events) were analyzed by flow cytometry. Apoptosis was determined by calculating the percentage of FITC^+^/PI^−^ cells (PI^+^ cells were considered necrotic cells).

### Cell cycle analysis

Cells were collected, washed with PBS and fixed with ice-cold 70% ethanol at −20°C for 1 day. After washing with PBS, cells were incubated with staining solution containing 10 μg/mL RNase (Sigma), 40 μg/mL propidium iodide and 0.1% NP-40 for 15 minutes. Fluorescence intensities of 10^4^ cells were measured using a FACS Calibur, and cell cycles were analyzed using FACSexpress Software.

### Western blotting

Cells were washed twice with cold PBS and lysed with ice-cold RIFA buffer (Cell Signaling). Lysates were centrifuged at 15, 000g (4°C) for 20 min to remove insoluble components, and protein concentrations in supernatants were measured using the BCA Protein Assay Kit (Thermo Scientific, Rockford, IL). Proteins (40 μg/lane) were separated on SDS-polyacrylamide gels and blotted onto nitrocellulose membranes (Whatman, Dassel, Germany). Western blotting was performed using antibodies against Akt, phospho-Akt, mTOR, phospho-mTOR, phospho-FAK, cyclin D1, phospho-Rb (Cell Signaling), p53, FAK, and β-actin (Santa Cruz Biotechnology, Paso Robles, CA). Membranes were developed using an enhanced chemiluminescence detection kit (Thermo Scientific).

### Immunofluorescence

MNCs were cultured on coverslips in 4-well plates. At day 4, cells were treated with deguelin (100 nM) or DMSO vehicle for 1.5 h and then fixed with 4% PFA. After washing and permeabilizing with 0.1% Triton X-100, cells were stained with Alexa Fluor 488-Phalloidin. DAPI was used to label nuclei.

### Immunohistochemistry

LLC xenograft tumors and mouse lungs were fixed in 4% PFA for 4 h, rinsed with PBS, and immersed in 30% sucrose in PBS overnight. Tissues were then placed in Tissue-Tek OCT Compound (Miles Inc., Elkhart, IN) at 4°C for 4 h before freezing with dry ice. Sections (8 μm) were then cut and stained with rat IgG anti-mouse CD31 antibody (BD Pharmingen) and Alexa Fluor 594-conjugated anti-rat IgG secondary antibody.

### Real-time PCR

Total RNA was extracted using a RNeasy Mini Kit (Qiagen, Valncia, CA) and reverse transcribed with a PrimeScript™ RT reagent Kit (Takara Bio Inc., Shiga, Japan). Real-time PCR was performed using an ABI PRISM 7300 Sequence Detection System using SYBR-Green PCR master mix (Applied Biosystems, Foster City, CA) with primer pairs targeting CXCR4 (forward 5′-GCCAAGTTCAAAAGCTCTGC-3′; reverse 5′-AGCCTCTGCTCATGGAGTTG-3′), VEGF (forward 5′-TCTCCCAGATCGGTGACAGT-3′; reverse 5′-GGGCAGAGCTGAGTGTTAGC-3′) and β-actin (forward 5′-AAGTCCCTCACCCTCCCAAAAG-3′; reverse 5′-AAGCAATGCTGTCACCTTCCC-3′). The mean cycle threshold (Ct) values from triplicate measurements were used to calculate relative gene expressions (normalized versus β-actin) using the ΔΔCt method.

### Statistical analysis

All data are expressed as the means ± SDs of at least 3 samples. ANOVA was used to determine the statistical significance of comparisons.

## SUPPLEMENTARY FIGURES



## Abbreviations

EPC: endothelial progenitor cell
BM: bone-marrow
CFU: colony forming units
MNC: mononuclear cell
ILK: integrin-linked kinase
FAK: focal adhesion kinase
GFP: green fluorescent protein
VEGF: vascular endothelial growth factor
SDF: strom cell-derived factor
ECFC: endothelial colony forming cells
EC: endothelial cells
PI3K: phosphatidylinositol 3-kinase
mTOR: mammalian target of rapamycin
Hsp: heat shock protein
HIF: hypoxia-inducible factor
KSL: c-kit+/sca-1+/Lin-
HUVEC: human umbellical vein endothelial cell
HDLEC: human dermal lympatic endothelial cell
eNOS: endothelial nitrogen oxide
