# Supplementary material for: Deguelin inhibits vasculogenic function of endothelial progenitor cells in tumor progression and metastasis via suppression of focal adhesion
Source: Oncotarget. 2015 Apr 27;6(18):16588–600. doi: 10.18632/oncotarget.3752 (PMC4599291; doi:10.18632/oncotarget.3752)
Supplement: Supplementary file 1 [file oncotarget-06-16588-s001.pdf]

SUPPLEMENTARY FIGURES

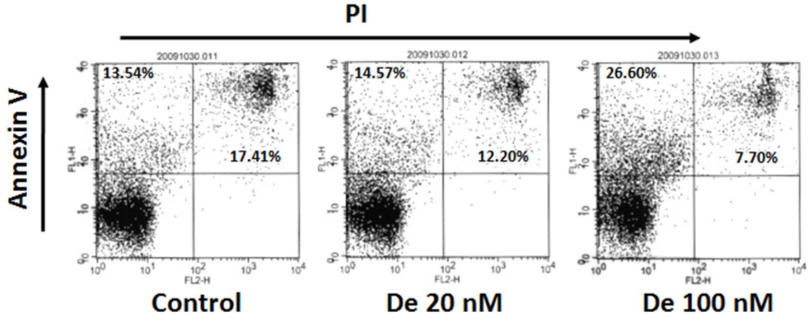

Supplementary Figure 1: Deguelin did not inhibit apoptosis in KSL cells. KSL cells were incubated with deguelin (0, 20, 100 nM) for 24 h in Stem span media. Cells were washed with PBS and stained with FITC-conjugated Annexin V and propidium iodide. Labeled cells were analyzed by flow cytometry.

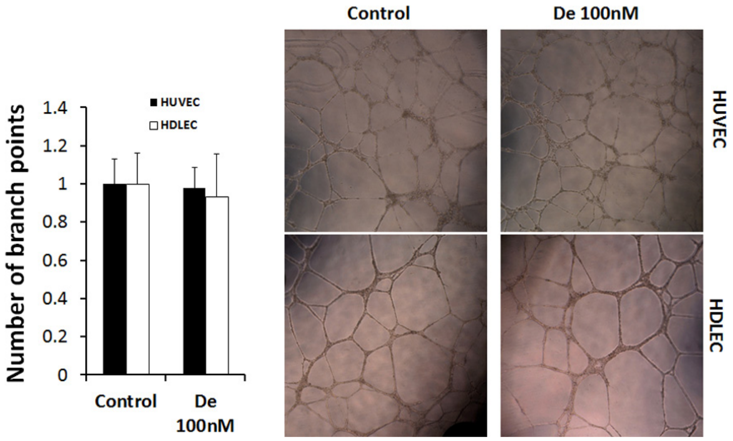

Supplementary Figure 2: Deguelin did not inhibit tube formation of HUVECs and HDLECs. HUVECs and HDLECs were cultured on Matrigel matrix for 8 h (n = 3). Representative tube-like structures are shown in right.

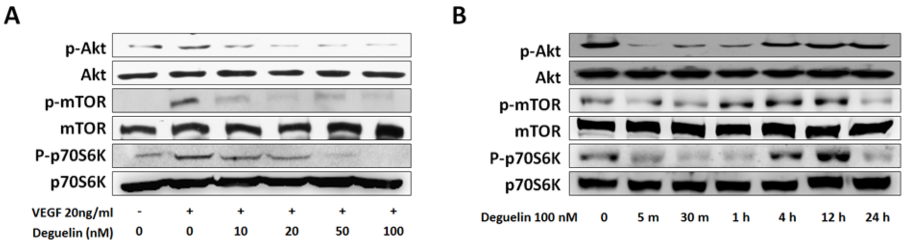

Supplementary Figure 3: Regulation of Akt-mediated pathways in EPCs by deguelin. A. Cultured EPCs were starved in serum-free EBM media for 24 h and then stimulated with rhVEGF (20 ng/ml) for 5 min. VEGF-induced phosphorylations of Akt (Ser473), mTOR, and p70S6K were detected by western blotting (n = 3). **B.** Cultured EPCs were treated with deguelin (100 nM). Levels of phosphorylated Akt, mTOR, and p70S6K were detected at different time points (n = 4).

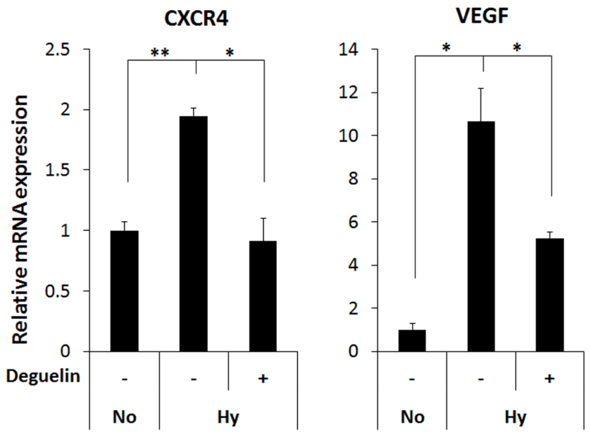

Supplementary Figure 4: Deguelin inhibited CXCR4 and VEGF expression in EPCs. Cultured EPCs were treated with deguelin (100 nM) under hypoxic conditions for 24 h. Levels of CXCR4 and VEGF mRNA were detected by real-time PCR and relative expression was calculated ($n = 3$).
